# Non-Linear Observer Design with Laguerre Polynomials

**DOI:** 10.3390/e24070913

**Published:** 2022-06-30

**Authors:** Maria Trigka, Elias Dritsas

**Affiliations:** Department of Computer Engineering and Informatics, University of Patras, 26504 Patras, Greece; trigka@ceid.upatras.gr

**Keywords:** non-linear dynamics, identifiability, observability, Laguerre polynomial

## Abstract

In this paper, a methodology for a non-linear system state estimation is demonstrated, exploiting the input and parameter observability. For this purpose, the initial system is transformed into the canonical observability form, and the function that aggregates the non-linear dynamics of the system, which may be unknown or difficult to be computed, is approximated by a linear combination of Laguerre polynomials. Hence, the system identification translates into the estimation of the parameters involved in the linear combination in order for the system to be observable. For the validation of the elaborated observer, we consider a biological model from the literature, investigating whether it is practically possible to infer its states, taking into account the new coordinates to design the appropriate observer of the system states. Through simulations, we investigate the parameter settings under which the new observer can identify the state of the system. More specifically, as the parameter θ increases, the system converges more quickly to the steady-state, decreasing the respective distance from the system’s initial state. As for the first state, the estimation error is in the order of $10^{-2}$ for θ=15, and assuming $c_{0}=\left\{0,1\right\},c_{1}=1$. Under the same conditions, the estimation error of the system’s second state is in the order of $10^{-1}$, setting a performance difference of $10^{-1}$ in relation to the first state. The outcomes show that the proposed observer’s performance can be further improved by selecting even higher values of θ. Hence, the system is observable through the measurement output.

## 1. Introduction

A common problem that many engineers have faced concerns the determination of the appropriate input so that the output has the desired behavior. However, before the control engineer starts designing the appropriate input, it should be investigated whether it is possible to achieve the desired behavior. A partial reply to the question of whether or not the appropriate control law exists is relevant to the investigation of whether the system is controllable or not. At the same time, for the implementation of the control law, a knowledge of state variables is essential. Therefore, an appropriate framework should be designed to determine the values of the variables. The design of such a structure (i.e., the controller) is feasible if the system is observable.

Moreover, in control theory, system identification constitutes a fundamental task for researchers and industrial professionals, and refers to the identification of a high-fidelity mathematical model that best describes the system. It is a solid way of describing the knowledge of a process or a system, and is extremely useful for all areas of science and technology, such as physics, chemistry, biology, engineering, economics, etc. [1]. The usefulness of a mathematical model lies in the fact that it gives information regarding the structure and properties of the system, while at the same time, it can help to predict and simulate its behavior. The problem of identification is usually very difficult, especially in those cases for which we do not have enough prior information regarding the system’s behavior [2]. For example, if the prior information for the system being identified is that it is linear, time-invariant (LTI), continuous-time, and with concentrated parameters, the model that describes the system is a differential equation with constant coefficients. On the other hand, if the system is non-linear and time-variant, the task becomes very complicated because the system model may be captured using ordinary differential equations (ODEs) with parameters that change over time. The system identifiability in both cases lies in determining the unknown parameters from the output measurements [2].

The analysis of identifiability can be made from a practical and structural perspective. The structural aims to identify the uncertainty, in parameter estimates thinking, of the deficiencies in the data used for model calibration. Additionally, structural identifiability is essential for practical identifiability and seeks to find whether the model parameters can be uniquely determined from observations of the input–output model [3]. Identifiability is intertwined with observability, which was first studied in systems and control theory, and describes the ability to infer the model states (namely, the dynamic variables described by differential equations in the model) from its output. The concept of structural identifiability (or, else, observability) was initially introduced in linear systems [4] and then extended to the nonlinear ones [5]. Similarly, the concept of observability was studied in nonlinear systems. Hence, the structural identifiability can be investigated with the aid of nonlinear observability tools [6].

In addition, the observability and structural identifiability [7] of a model can be simultaneously studied by including the unknown parameters in the state variables vector and calculating the rank of the augmented observability matrix. This is similar to the limitation of the differential algebra approach to the initial conditions. Hence, as the system evolves from an initial state, it may be impossible to identify it (e.g., to determine some of its parameters) if the observability matrix is rank-deficient [1].

In control theory, identification and identifiability are mostly related to observer design and observability. For a dynamic system described in the state space, the estimation of the state vector can be made on the basis of inputs and outputs, while, alternatively, it can be made by designing a dynamic system called the observer. In order to be able to determine the initial state, the observability matrix is enough to be invertible. This implies that it will be of full rank, which constitutes the algebraic criterion for the observability [8].

In this research article, the structural properties of nonlinear systems and preliminaries on observability are investigated, exploiting, from the differential geometry, the Lie derivative to develop the Observability Rank Condition (ORC) [9]. Our aim is to study the identifiability of a nonlinear system exploiting the “Canonical Observability” form and the Laguerre polynomials to model the unknown non-linearities through a function Φ(·) that occurs in this representation. The identification is equivalent to the estimate of the unknown parameters, which subsequently leads to the estimation of the state vector. The choice of polynomials lies in the fact they can form an orthonormal basis where each representation of the state vector in this space can be expressed as a linear combination of these base functions. The identification is executed through observation, and the observer that is constructed evaluates the state vector, and at the same time, identifies the system. Here, the unknown parameters are the coefficients that multiply the Laguerre polynomials. The above procedure is useful if the state space equations that describe the dynamics of the system, are unknown (partially or full), as long as the system is observable for each input, and its output is measurable.

The paper is organized as follows. Section 2 presents the necessary background around the concept of observability. Next, Section 3 shows the problem formulation of the proposed approach for the identification of a non-linear system. Section 4 demonstrates the evaluation of the elaborated approach, considering an application example from biology and experimenting with the involved parameters. Finally, Section 5 summarizes the main findings of the research study and sets future directions.

## 2. Materials and Methods

The observability of a system is an important concept in modern control theory, and was introduced by Kalman [10]. Observability is a structural property of control systems, and is related to the ability to determine the initial state of the system from the input and output measurements. Hence, this section introduces and defines the types of observability for the state of the system and the system as a whole entity. Additionally, the given definitions hold for both linear and non-linear systems. In the same section, we determine the observability matrix and the related conditions that should be satisfied, such that a system (either linear or nonlinear) is observable. In the following, a list of designations is summarized in Table 1, and definitions will be given before the presentation of the proposed approach.

### 2.1. Notations and Definitions

To define the observability, it is necessary to introduce the notion of distinguishable states. In addition, we considered nonlinear systems of input u(t) and output y(t), which may be written in the following form:

(1) $$\left\{\begin{array}{cc} \dot{x}\left(t\right)=g\left(x\left(t\right),u\left(t\right)\right) & x\left(t_{0}\right)=x_{0},x\in M:=R^{n}\\ y(t)=h(x(t)) & u(t)\in \Omega ,t\in [0,T] \end{array}\right.$$

**Definition** **1.** *Let a model with an internal state x and a measurable output y. Let $y_{x_{0}}$ denote the time evolution of the model output when started from an initial state $x_{0}$ at $t_{0}$. $x_{1}$ and $x_{2}$ are indistinguishable if $y_{x_{1}}=y_{x_{2}}$ for all $t\geq t_{0}$. The set of states that are indistinguishable from $x_{1}$ is denoted by $I(x_{1})$.*

**Definition** **2.** ***Observable State:** A state $x_{1}$ is observable if the set of states that are indistinguishable from $x_{1}$ include only $x_{1}$, e.g., $I\left(x_{1}\right)=\left\{x_{1}\right\}$, ∀u,y. A state is observable if there is at least one input for which the corresponding output is distinguishable from every other state.*

**Definition** **3.** ***Observable System:** A system is observable if each state of the system is observable, e.g., ∀x∈M, I(x)={x}.*

Because observability is a more general concept, Hermann et al. [11] first defined the so-called local and local weak observability. These definitions were also adopted by [5]. Here, we briefly remind the reader of the definitions and their practical meaning.

**Definition** **4.** *Two states $x_{1},x_{2}\in M$ are called M-indistinguishable in [0,T] if: $x_{1}I_{M}x_{2}↔y_{x_{1}}=y_{x_{2}}$, $\forall t\in \left[0,T\right],\forall u\in \Omega ,\;y_{x_{1}},y_{x_{2}}\in M$, denoted by $x_{1}I_{M}x_{2}$. A state $x_{1}$ is distinguishable from a state $x_{2}$ if ∀u the courses of $x_{1},x_{2}\in M$ and $y_{x_{1}},y_{x_{2}}$ are indistinguishable.*

**Remark** **1.** *The set of M-indistinguishable points is denoted as: $I\left(x_{1}\right)\overset{\Delta }{=}\left\{x_{1}\in M|x_{1}I_{M}x_{2}\right\}$. The M-observable state is $I\left(x_{1}\right)\overset{\Delta }{=}\left\{x_{1}\right\}$.*

**Definition** **5.** ***Locally Observable State:** A state $x_{1}$ is locally observable if, for every open neighborhood U of $x_{1}$, the set of states that are indistinguishable from $x_{1}$ include only $x_{1}$, e.g., $I\left(x_{1}\right)=\left\{x_{1}\right\}$.*

**Definition** **6.** ***Weakly Observable System:** A system is locally observable in [0,T] if each state is locally observable.*

**Definition** **7.** ***Locally Weakly Observable State:** A state $x_{1}$ is locally weakly observable if there is an open neighborhood U of $x_{1}$ s.t. for each open neighborhood V of $x_{1}$ with V⊂U (included into M), the set of states of V that are indistinguishable from $x_{1}$ include only $x_{1}$, e.g., $I_{V}\left(x_{1}\right)\cap M=\left\{x_{1}\right\},\forall V$.*

**Definition** **8.** ***Locally Weakly Observable System:** A system is locally weakly observable in [0,T] if its state is locally and weakly observable.*

**Definition** **9.** The state $x_{0}\in R^{n}$ of a system is observable in the time moment $\tau_{0}$ if there is a finite $\tau^{*}>\tau_{0}$, such that $x_{0}$ can be determined by $y(\tau_{0},\tau^{*})$ and $u(\tau_{0},\tau^{*})$.

**Definition** **10.** The state $x_{0}\in R^{n}$ of a system is observable if $\forall \tau_{0}$ there is a finite $\tau^{*}>\tau_{0}$ such that $x_{0}$ can be determined by $y(\tau_{0},\tau^{*})$ and $u(\tau_{0},\tau^{*})$.

**Definition** **11.** The system is observable at $\tau_{0}$ if given $\tau_{0}$ there is a finite $\tau^{*}>\tau_{0}$ such that $x_{0}$ can be determined by $y(\tau_{0},\tau^{*})$ and $u(\tau_{0},\tau^{*})$.

### 2.2. Observability and Non-Linear Systems

The study of observability in nonlinear systems is a more difficult task, and for this reason, we should exploit more advanced mathematical methods from differential geometry, such as the Lie derivative (i.e., the derivative in the direction of a vector field). In non-linear systems, the observability depends on the system input(s); thus, an observer would operate rationally only if the system is observable for the specific input(s). Nonetheless, especially for the non-linear systems, the existence of a canonical form is analogous to mentioning that the system is observable for any input.

If we know the output derivative, we can use a direct method of calculating the state vector for the study of observability in nonlinear systems. Assuming the vector field $f:R^{n}\to R^{n}$ and $h:R^{n}\to R^{n}$ a smooth function (namely, differentiable), the first-order Lie derivative of the output, with respect to the vector field, is defined as follows [12]:

(2) $$L_{f}\left(h\left(x\right)\right)=\frac{\partial h(x)}{\partial x}f=\sum_{i=1}^{n}\frac{\partial h(x)}{\partial x_{i}}f_{i}\left(x\right)=\nabla h\left(x\right)f\left(x\right),$$

where $f\left(x\right)={\left[\begin{array}{ccc} f_{1}\left(x\right), & f_{2}\left(x\right), & \ldots f_{n}\left(x\right) \end{array}\right]}^{T}$, $\nabla h\left(x\right)=\left[\begin{array}{ccc} \frac{\partial h(x)}{\partial x_{1}} & \frac{\partial h(x)}{\partial x_{2}} & \cdots \frac{\partial h(x)}{\partial x_{n}} \end{array}\right]$, and by definition, $L_{f}^{0}\left(h\left(x\right)\right)=h\left(x\right)$. The second-order derivative is $L_{f}^{2}\left(f\left(x\right)\right)=\frac{\partial L_{f}\left(h\left(x\right)\right)}{\partial x}h\left(x\right)$, and the higher-order Lie derivatives are defined accordingly.

The observability matrix O is defined as $O\left(x\right)=\frac{\partial }{\partial x}\left[\begin{array}{c} L_{f}^{0}\left(h\left(x\right)\right)\\ L_{f}\left(h\left(x\right)\right)\\ \vdots \\ L_{f}^{n-1}\left(h\left(x\right)\right) \end{array}\right]$. To be the system observable, its rank (O) should be equal to *n*.

If the input signal of a system is a time-constant function, then we can say that we have an autonomous (or state-affine) system of the form:

(3) $$\begin{array}{c} \dot{x}=f\left(x\right),x={\left[x_{1},x_{2},\ldots ,x_{n}\right]}^{T}\\ y=h(x)\in R. \end{array}$$

It is evident that for the study of observability, state-affine systems are equivalent to linear time-varying systems.

For an LTI dynamic system represented as:

(4) $$\begin{array}{c} \dot{x}\left(t\right)=Ax\left(t\right)+Bu\left(t\right)\\ y(t)=Cx(t). \end{array}$$

To infer the complete initial state $x(t_{0})$ uniquely, a relationship between the outputs y(t), the state vector x(t), and the inputs u(t) should be established. This relationship is captured by the observability matrix, adopting a similar algebraic criterion as the one for non-linear dynamical systems, stating that (3) is observable if the rank, namely, $rank\left(O\right)=rank\left({\left[\begin{array}{ccccc} C & CA & CA^{2} & \ldots & CA^{n} \end{array}\right]}^{T}\right)$ is equal to *n*.

## 3. Proposed Approach

Treating a system as a black box, the signals that are usually directly accessible are the input and output. In many cases, to change the dynamics of the system or to access its behavior, it is necessary to know the characteristics of the system; i.e., the state vector. Hence, the need to design an observer system in order to obtain access to state variables is raised. Here, we will study the problem of constructing a state observer for one autonomous nonlinear system of the form:

(5) $$\begin{array}{c} \left(\Sigma \right):\dot{x}=f\left(x\right),\\ y=h\left(x\right),x\in M\subset R^{n} \end{array}$$

where, in practical scenarios, Ω is an open, connected, and relatively compact set that constitutes the state space of the system. In ideal conditions, it may be positive invariant under the system dynamics.

The system (Σ) is observable in *M* if the output data in one finite period of time $\left[t_{0},t_{1}\right]$, where $t_{1}>t_{0}$, fully defines the initial state $x(t_{0})$. (At least for trajectories x(t), such that x(t)∈M for any $t\in [t_{0},t_{1}]$.) The fact that system (Σ) is observable is equivalent to the requirement that the set of functions $O\left(\Sigma \right)=\{L_{f}^{0}\left(h\left(x\right)\right),L_{f}\left(h\left(x\right)\right),\ldots ,L_{f}^{i}\left(h\left(x\right)\right)\}$, i≥0, called its “observability space”, separates *M* points, i.e., such that $\forall x_{1},x_{2}\in \Omega$ exists *i* for which $L_{f}^{i}\left(h\left(x_{1}\right)\right)=L_{f}^{i}\left(h\left(x_{2}\right)\right)$.

### 3.1. Problem Formulation

Here, we will see how a nonlinear system can be written in the “Observable Canonical Form” of the Gauthier-Hammouri-Othman [13,14]. The canonical form is the one in which a nonlinear system can be transformed, ensuring that it is observable for any input. Conversely, in a nonlinear system that is in canonical form, we can assume with certainty that it is observable for each input. The canonical form is important, since we will rely on it to study the construction of a nonlinear state observer. When the system is observable the differentiation is almost normal everywhere.

(6) $$\left\{\begin{array}{c} F_{\Sigma }:R^{n}\to R^{n}\\ x\to \left[\begin{array}{c} h(x)\\ L_{f}\left(h\left(x\right)\right)\\ \vdots \\ L_{f}^{n-1}\left(h\left(x\right)\right) \end{array}\right] \end{array}\right.$$

Assuming the new variables $z_{i}=L_{f}^{i-1}\left(h\left(x\right)\right),i=1,2,\ldots ,n$, the output function of the original system is $y=h\left(x\right)=s=z_{1}$, and so, the new variables are related to the old ones according to the following relationships:

(7) $$\dot{z}=\left[\begin{array}{c} {\dot{z}}_{1}\\ {\dot{z}}_{2}\\ \vdots \\ {\dot{z}}_{n-1}\\ {\dot{z}}_{n} \end{array}\right]=\left[\begin{array}{c} z_{2}\\ z_{3}\\ \vdots \\ z_{n-2}\\ \Phi (z) \end{array}\right]=F\left(z\right)$$

where F(·) is the function of the canonical form. So, the state observer of the system, which is written in canonical observability form, will be a system that is described as:

(8) $$\dot{\hat{z}}=F\left(\hat{z}\right)-S^{-1}C^{T}\left(Cy-\hat{z}\right)$$

with S being the solution of the Riccati equation in the steady state:

(9) $$0=-\theta S-A^{T}S-SA+C^{T}C$$

with $A:R^{n}\to R^{n},A_{i,j}=\delta_{i,j-1}$, C=[1,0,0,…,0,0]. We find the observer equations based on the canonical observability form, and based on these going backwards, we find the observer equations for the initial state variables. So, we construct an observer for a nonlinear system. The system observer will converge [15] according to the relation:

(10) $$∥x\left(t\right)-\hat{x}\left(t\right)∥=Ke^{-\theta t/3}∥x_{0}-{\hat{x}}_{0}∥.$$

The parameter θ affects the speed convergence of the observer. The larger the θ, the faster the observer will converge.

In order to identify the system, it is enough to identify Φ. This function is generally nonlinear, and there is no other information that could help with its identification. The proposed approach, which is the main contribution of this work, is to represent Φ by using the Laguerre polynomials. This is essentially the way of identification that will be presented in the current study. Laguerre polynomials have been utilized in identification tasks in order to capture the dynamic behavior of real systems [16]. Here, these polynomials are exploited to model the first-order derivative of the n-th variable $z_{n}$ which is involved in the canonical observability form in (7). Because these polynomials meet the orthogonality properties, they can constitute the basis of a space where every function in that space can be expressed as a linear combination of Laguerre polynomials, hence, $\Phi \left(z\right)=\sum_{i=1}^{n}c_{i}{∥L_{i}\left(z\right)∥}_{2}$. The system identification lies in the estimation of appropriate $c_{i}$. The estimations of these coefficients could be achieved using the Extended Kalman filter. The Kalman filter will essentially be the nonlinear state observer of the system, which will estimate the state vector and, at the same time, the unknown parameters. Here, exploiting the polynomial Laguerre, we approximate the initial Φ(z) with a new function which is still a polynomial, and thus, differentiable in terms of the system states.

We consider the simple Laguerre polynomials, which are the special case a=0 of the generalized Laguerre polynomials, $L_{n}^{\left(0\right)}\left(z\right)=L_{n}\left(z\right)$. The Laguerre polynomials constitute the solution of the second-order differential (Laguerre) equation: [17]

(11) $$zL_{n}^{\prime \prime }\left(z\right)+\left(1-z\right)L_{n}^{\prime }\left(z\right)+nL_{n}\left(z\right)=0,$$

which are defined, based on Rodrigues’ formula, as $L_{n}\left(z\right)=e^{z}\frac{\partial^{n}\left(z^{n}e^{-z}\right)}{\partial^{n}z}$. Assuming n=3, $L_{0}\left(z\right)=1,L_{1}\left(z\right)=-z+1,L_{2}\left(z\right)=0.5z^{2}-2z+1$. The orthonormal conditions of the Laguerre polynomials (with respect to the weighting function $e^{-z}$) are [18,19]:

(12) $$\left\{\begin{array}{c} \int_{0}^{\infty }e^{-z}L_{n}\left(z\right)L_{m}\left(z\right)\;dz=0,m\neq n\\ \int_{0}^{\infty }e^{-z}L_{n}\left(z\right)L_{m}\left(z\right)\;dz={\left(n!\right)}^{2},m=n. \end{array}\right.$$

Given the proposed Φ, which is differentiable (i.e., it satisfies the Lipschitz condition [20]), we aim to design an observer whose error dynamic is asymptotically stable.

### 3.2. Bioreactor States Estimator Design

In this section, we present how the previous analysis can be applied for the estimation of the state of bioreactors. For this purpose, the biological processes’ state-space model is captured and then utilized for the estimators’ design. The process is assumed to be continuous with a dilution rate, *D* and an input substrate concentration, $s_{0}$. We notice here that the *D* is a scalar.

More specifically, the biological reactor processes are described by the following model [21,22,23], in the state space:

(13) $$\begin{array}{c} \left\{\begin{array}{c} \dot{s}\left(t\right)=D\left(t\right)\left(s_{0}-s\left(t\right)\right)-\frac{\mu (s(t))x(t)}{R}\\ \dot{x}\left(t\right)=\left(\mu \left(s\left(t\right)\right)-D\left(t\right)\right)x\left(t\right)\\ y(t)=s(t). \end{array}\right. \end{array}$$

The variables s(t) and x(t) are the concentration of biomass (kg/m${}^{3}$) and substrate (kg/m${}^{3}$), respectively, and $\mu_{max},R,\;\mathrm{and}\;K$ are the characteristic parameters of the process. The main assumption concerning the process is that cell growth is described by the Mond rule, $\mu \left(s\left(t\right)\right)=\mu_{max}\frac{s(t)}{K+s(t)}$ with $\mu_{max}$ being the maximum specific growth rate and *K* being a saturation constant. Additionally, the cell death is negligible as to the rate of the cell growth. By the Monod kinetics rule [24], the equations are rewritten as:

(14) $$\begin{array}{c} \left\{\begin{array}{c} \dot{s}\left(t\right)=D\left(s_{0}-s\left(t\right)\right)-\frac{\mu_{max}}{R}\frac{s(t)x(t)}{K+s(t)}\\ \dot{x}\left(t\right)=\left(\mu_{max}\frac{s(t)}{K+s(t)}-D\right)x\left(t\right)\\ y(t)=s(t) \end{array}\right. \end{array}$$

We assume that there is no biomass in the feed stream; thus, $x_{0}=x\left(0\right)=0$.

In [25], necessary and sufficient conditions have been derived for the observability of models of biochemical processes. Additionally, Hammouri et al., in [26], proposed a practical method for constructing an observer based on the immersion of autonomous nonlinear systems into a nonlinear form of observability. A similar process will be followed to build the suggested approach. More specifically, to transit into the new coordinates system, the following transformation is adopted:

(15) $$\begin{array}{c} \left\{\begin{array}{c} z_{1}=h\left(x\right)=s\left(t\right)\\ z_{2}=L_{f}\left(h\left(x\right)\right)=\dot{s}\left(t\right)=D\left(s_{0}-s\left(t\right)\right)-\frac{\mu_{max}}{R}\frac{s(t)}{K+s(t)}x\left(t\right) \end{array}\right. \end{array}$$

where the variables $z_{1},z_{2}$ constitute the new states of the system. The relationship between the initial and new states are as follows:

(16) $$\begin{array}{c} \left\{\begin{array}{c} s\left(t\right)=z_{1}\\ x\left(t\right)=-R\frac{K+z_{1}}{\bar{\mu }}\left(z_{2}+Dz_{1}-Dz_{10}\right) \end{array}\right. \end{array}$$

with $z_{10}=s_{0}$.

To represent the initial system in the state space “Canonical Form”, we exploit Equation (13), from which we derive the first-order time derivatives of $z_{1},z_{2}$. Hence, the acquired system is written as:

(17) $$\begin{array}{c} \left\{\begin{array}{c} {\dot{z}}_{1}=z_{2}\\ {\dot{z}}_{2}=\Phi \left(z_{1},z_{2}\right)\\ y=z_{1} \end{array}\right. \end{array}$$

In (17), $\Phi \left(z_{1},z_{2}\right)=\frac{Kz_{2}+\bar{\mu }z_{1}^{2}+Dz_{1}\left(K+z_{1}\right)}{\left(K+z_{1}\right)z_{1}}\left(z_{2}+Dz_{1}-Dz_{10}\right)$ is the non-linear full Lipschitzian function in $R^{2}$. Therefore, the system is uniformly observable (i.e., for each input $z_{10}$, or equivalently, $s_{0}$) and the initial state may be reconstructed using the input and measurement data. It is obvious that the system in (15) is observable when:

(18) $$rank\left(O\right)=rank\left(\left[\begin{array}{cc} \frac{\partial h(x))}{\partial s} & \frac{\partial h(x))}{\partial x}\\ \frac{\partial L_{f}\left(h\left(x\right)\right)}{\partial s} & \frac{\partial L_{f}\left(h\left(x\right)\right)}{\partial x} \end{array}\right]\right)=rank\left(\left[\begin{array}{cc} 1 & 0\\ \frac{\partial \dot{s}}{\partial s} & -\frac{\mu_{max}s}{R(K+s)} \end{array}\right]\right)=2,$$

which is satisfied if $det\left(O\right)=\frac{\mu_{max}s}{R(K+s)}\neq 0$. To design the observer, we exploited (6) and (7), where the steady-state solution of the Riccati equation [15] is $S=\left[\begin{array}{cc} \frac{1}{\theta } & -\frac{1}{\theta^{2}}\\ -\frac{1}{\theta^{2}} & \frac{2}{\theta^{3}} \end{array}\right]$, which holds that $S=S^{T}$ and $S^{-1}=\left[\begin{array}{cc} 2\theta & \theta^{2}\\ \theta^{2} & \theta^{3} \end{array}\right]$, with $A=\left[\begin{array}{cc} 0 & 1\\ 0 & 0 \end{array}\right]$, $C=\left[\begin{array}{cc} 1 & 0 \end{array}\right]$. Then, the observer is captured as:

(19) $$\begin{array}{c} \left\{\begin{array}{c} {\dot{\hat{z}}}_{1}={\hat{z}}_{2}-2\theta \left({\hat{z}}_{1}-z_{1}\right)\\ {\dot{\hat{z}}}_{2}=\Phi \left(z_{1},z_{2}\right)-\theta^{2}\left({\hat{z}}_{1}-z_{1}\right) \end{array}\right. \end{array}$$

In order to design the proposed observer, we represent $\Phi (z_{1},z_{2})$ as:

(20) $$\begin{array}{c} \Phi \left(z_{1},z_{2}\right)=c_{0}{∥L_{0}\left(z\right)∥}_{2}+c_{1}L_{1}\left(z\right){∥}_{2}\\ =c_{0}\sqrt{2}+c_{1}\sqrt{{\left(1-z_{1}\right)}^{2}+{\left(1-z_{2}\right)}^{2}}, \end{array}$$

where $L_{0}\left(z\right)={\left[\begin{array}{cc} 1 & 1 \end{array}\right]}^{T}$, $L_{1}\left(z\right)={\left[\begin{array}{cc} 1-z_{1} & 1-z_{2} \end{array}\right]}^{T}$.

Going backwards, the observer equations for the state variables of the initial system will be:

(21) $$\begin{array}{c} \left\{\begin{array}{c} \dot{\hat{s}}\left(t\right)=D\left(s_{0}-\hat{s}\left(t\right)\right)-\frac{\mu_{max}}{R}\frac{\hat{s}\left(t\right)}{K+\hat{s}\left(t\right)}\hat{x}\left(t\right)-2\theta \left(\hat{s}\left(t\right)-s\left(t\right)\right)\\ \dot{\hat{x}}\left(t\right)=-\frac{R}{\mu_{max}}\frac{K+\hat{s}\left(t\right)}{\hat{s}\left(t\right)}(\left(\frac{\mu_{max}}{R}\frac{\hat{s}\left(t\right)}{K+\hat{s}\left(t\right)}+D\right)\left(D\left(s_{0}-\hat{s}\left(t\right)\right)-\frac{\mu_{max}}{R}\frac{\hat{s}\left(t\right)}{K+\hat{s}\left(t\right)}\hat{x}\left(t\right)\right)+\\ c_{0}\sqrt{2}+c_{1}\sqrt{{\left(1-\hat{s}\left(t\right)\right)}^{2}+{\left(1-D\left(s_{0}-\hat{s}\left(t\right)\right)+\frac{\mu_{max}}{R}\frac{\hat{s}\left(t\right)}{K+\hat{s}\left(t\right)}\hat{x}\left(t\right)\right)}^{2}}\\ -\theta^{2}\left(\hat{s}\left(t\right)-s\left(t\right)\right)) \end{array}\right. \end{array}$$

At this point, it should be noticed that, from (8), the observer of (14) (without the application of the canonical form) is formulated as:

(22) $$\begin{array}{c} \left\{\begin{array}{c} \dot{\hat{s}}\left(t\right)=D\left(s_{0}-\hat{s}\left(t\right)\right)-\frac{\mu_{max}}{R}\frac{\hat{s}\left(t\right)\hat{x}\left(t\right)}{K+\hat{s}\left(t\right)}-2\theta \left(\hat{s}\left(t\right)-s\left(t\right)\right)\\ \dot{\hat{x}}\left(t\right)=\left(\mu_{max}\frac{\hat{s}\left(t\right)}{K+\hat{s}\left(t\right)}-D\right)\hat{x}\left(t\right)-\theta^{2}\left(\hat{s}\left(t\right)-s\left(t\right)\right) \end{array}\right. \end{array}$$

The observer’s models in (21) and (22) will be evaluated in the experiments.

## 4. Results and Discussion

The experiment environment has the following characteristics: An Intel(R) Core(TM) i7-9750 H CPU @ 2.60 GHz 2.59 GHz 16 GB memory, Windows 10 Home, 64-bit operating system, ×64-based processor, and MATLAB 2022. In this section, we attempt to illustrate the reconstruction ability of the proposed observer in a realistic scenario. In order to illustrate the performance of the proposed observer–estimator, we present the results of the process Models (14) and (21). The simulation of the observers’ models was performed under the following parameters settings: $x\left(0\right)=0\;g/l,s\left(0\right)=12.0\;g/l,\mu_{max}=0.2,R=0.3$. The experiments aim to show the impact of parameter θ and the unknown parameters $c_{0},c_{1}$ on the estimate of the initial state of the bioreactor process. Notation O1 refers to the observer of (14), while O2 relates to the proposed observer, which is captured by (21).

**Figure 1:**
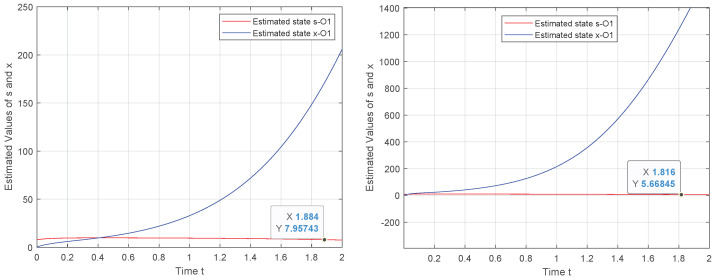
Observer 1 behavior for $\theta =\left\{5,15\right\},x\left(0\right)=\left[\begin{array}{c} 8\\ 0.5 \end{array}\right]$.

In Figure 1, the performance of O1 is illustrated for two different values of the convergence parameter θ. We see that when the observer is designed based on the state–space model captured in (10), the initial state of the system is not successfully estimated. This behavior relates to the fact that the observability is not satisfied.

**Figure 2:**
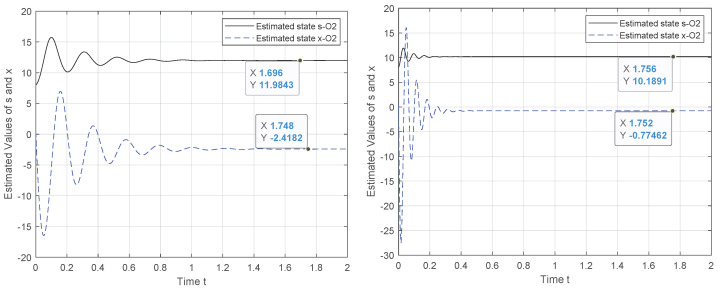
Observer behavior for θ={5,15}, $c_{0}=c_{1}=4,x\left(0\right)=\left[\begin{array}{c} 8\\ 0.5 \end{array}\right]$.

**Figure 3:**
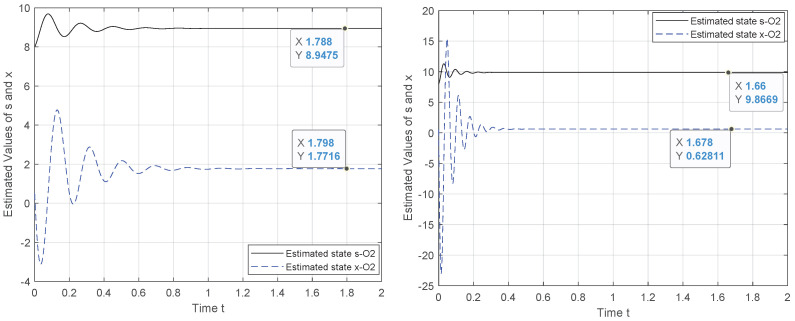
Observer behavior for θ={5,15}, $c_{0}=-c_{1},c_{1}=4,x\left(0\right)=\left[\begin{array}{c} 8\\ 0.5 \end{array}\right]$.

**Figure 4:**
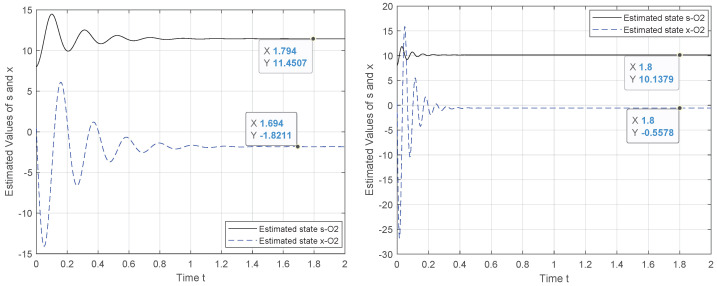
Observer behavior for θ={5,15}, $c_{1}=-c_{0},c_{0}=4,x\left(0\right)=\left[\begin{array}{c} 8\\ 0.5 \end{array}\right]$.

**Figure 5:**
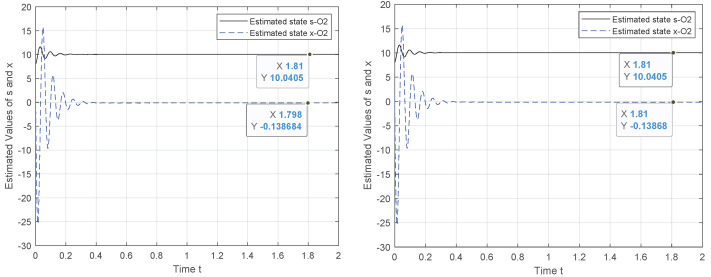
Observer behavior for θ={15}, $c_{0}=\left\{0,1\right\},c_{1}=1,x\left(0\right)=\left[\begin{array}{c} 8\\ 0.5 \end{array}\right]$.

To tackle this weakness, we exploit the canonical observability form to design a new observer system for which the observability holds. Hence, the results in Figure 2, Figure 3, Figure 4 and Figure 5 demonstrate the performance of the proposed observer whose behavior is represented by (21). The experiment outcomes reveal the capability of the suggested observer to track the initial state of the system whose speed convergence increases with the increase in θ, verifying the role of this parameter, as indicated by (10). Moreover, the performance of the suggested observer is evaluated by selecting different pairs $\left(c_{0},c_{1}\right)$ of the unknown parameters involved in the new representation of function Φ(·).

The initial state reconstruction is evaluated, assuming a noiseless scenario under various selections for the unknowns’ parameters. The results verify that the recovery of the initial state is achieved with satisfactory accuracy. As illustrated in Figure 4 and Figure 5, when the two unknown parameters are configured to $(c_{0},c_{1})=$(1,1) or (0,1), the initial state of *s* attained the measured output y=s, although for the problem solution, we consider that the system starts from a random state, $x\left(0\right)=\left[\begin{array}{c} 8\\ 0.5 \end{array}\right]$.

The output measures only the first state, s(t), which, as we see, may underestimate the unmeasured state, x(t), as well as the two unknown parameters, $c_{0}$ and $c_{1}$, which participate in the modeling of Φ(·). The lower (but still acceptable) estimation accuracy of these quantities is justified by their correlation that prevents their perfect identification. The methodology achieves good reconstruction without the use of any prior knowledge.

## 5. Conclusions

This paper focuses on nonlinear observability definitions with an emphasis on geometrical and algebraic tools. Moreover, it presents a methodology for the transformation of a nonlinear system into Observability Canonical Form, which facilitates the establishment of an observer that is suitable for the estimation of the initial state of the system. In exploiting the new coordinates system, it suffices to know the first state s(t) (which should also be differentiable) of the system in order to effectively estimate its states. From the evaluation of the current methodology, we conclude that the system representation in canonical form and the modeling of the second state of the system as a linear combination of the Laguerre polynomials could lead to the identification of the initial state of the system, even if we have partial knowledge of its dynamics.

Moreover, we observe that in the steady-state, the first state *s* is observable by the output. Additionally, as the parameter θ increases, the system converges more quickly to the steady-state, as dictated by theory (10). As for the first state, the absolute deviation of the estimated from the true initial state is on the order of $10^{-2}$ for θ=15, and assuming $c_{0}=\left\{0,1\right\},c_{1}=1$. However, considering $c_{0}=c_{1}\neq 1$, $c_{0}=-c_{1}$, $c_{1}=-c_{0}$, the error is on the order of $10^{-1}$. Similar promising behavior is observed for the second state of the system, with an error on the order of $10^{-1}$, which seems to decrease and attain a value that is more close to zero for θ=15 (and even higher). Finally, we see that the performance difference in the estimate of s,x is one order of magnitude.

In future work, we aim to study other basis functions that are suitable for the modeling of the unknown dynamics Φ(·) of the system in the canonical form, and evaluate their capability under the same objective. Finally, a challenging direction would be to experiment with other case studies such as the HIV and glucose dynamics models, in order to design a proper control algorithm for a future closed-loop therapy system. 

## Figures and Tables

**Table 1:** List of Designations.

Notation	Description
*M*	state space, an open set on $R^{n}$
U	subset of *M*
x∈M	system state
$\hat{x}$	estimated state
u	system input
h(x)	system output ∈R
$x_{0}$	initial state at $t_{0}$
$\dot{x}$	derivative of x with respect to the time variable t
$y_{x_{1}}$	output at state $x_{1}$
$x_{1}I_{M}x_{2}$	$x_{1},x_{2}$*M*- indistinguishable
O	observability matrix
${∥x∥}_{2}$	the vector 2-norm (the Euclidean norm)
$L_{f}^{i}\left(\cdot \right)$	Lie derivative i-th order
Φ(x)	a nonlinear function that is continuous with respect to x
